# Traditional, complementary, and integrative medicine evidence map: a methodology to an overflowing field of data and noise

**DOI:** 10.26633/RPSP.2021.48

**Published:** 2021-04-23

**Authors:** Mariana Cabral Schveitzer, Carmen Verônica Mendes Abdala, Caio Fabio Schlechta Portella, Ricardo Ghelman

**Affiliations:** 1 Universidade Federal de São Paulo São Paulo Brazil Universidade Federal de São Paulo, São Paulo, Brazil; 2 PAHO/WHO Latin American and Caribbean Center on Health Sciences Information (BIREME) São Paulo Brazil PAHO/WHO Latin American and Caribbean Center on Health Sciences Information (BIREME), São Paulo, Brazil; 3 Brazilian Academic Consortium for Integrative Health (CABSIN) São Paulo Brazil Brazilian Academic Consortium for Integrative Health (CABSIN), São Paulo, Brazil

**Keywords:** Systematic review, complementary therapies, integrative medicine, coronavirus infections, Revisión sistemática, terapias complementarias, medicina integral, infecciones por coronavirus, Revisão sistemática, terapias complementares, medicina integrativa, infecções por coronavirus

## Abstract

Every day there is criticism about lack of evidence on traditional, complementary, and integrative medicine (TCIM). But is this narrative evidence-based? Are we really missing research about TCIM? Or are we just not looking correctly at the evidence? Evidence maps are a useful method with the dual function of synthesizing available evidence on a specific topic and identifying knowledge gaps. This article presents a six-step evidence map methodology along with recently published TCIM evidence maps, including one related to COVID-19. TCIM evidence maps are useful instruments to inform decision-making for policymakers, health practitioners, and patients.

## RESUMEN

Es habitual que se critique la falta de evidencia con respecto a las medicinas tradicionales, complementarias e integrativas. Sin embargo, ¿se basa en la evidencia este discurso? ¿Falta realmente investigación sobre las medicinas tradicionales, complementarias e integrativas o es que simplemente no estamos analizando la evidencia de forma adecuada? Los mapas de evidencia son un método útil que tiene una función doble: sintetizar la evidencia disponible por temas específicos y determinar si hay alguna laguna en el conocimiento. En este artículo se presenta una metodología de elaboración de mapas de la evidencia en seis pasos, junto con los mapas de la evidencia de las medicinas tradicionales, complementarias e integrativas publicados recientemente, incluido un mapa sobre la COVID-19. Los mapas de la evidencia de las medicinas tradicionales, complementarias e integrativas son instrumentos útiles para fundamentar la toma de decisiones por parte de los encargados de las políticas, el personal de salud y los pacientes.

## RESUMO

A crítica é diária de que faltam evidências em medicinas tradicionais, complementares e integrativas (MTCI). Mas será que esta narrativa se baseia em evidências? Realmente faltam pesquisas em MTCI? Ou será que simplesmente não estamos atentando corretamente às evidências? Os mapas de evidências consistem em uma metodologia útil de dupla função: sintetizar as evidências existentes em um determinado tópico e identificar as lacunas de conhecimento. Neste artigo é apresentada uma metodologia de mapa de evidências de seis passos junto com mapas de evidências de MTCI recém-publicados, incluindo um relacionado à COVID-19. Os mapas de evidências de MTCI são instrumentos úteis para subsidiar a tomada de decisão dos responsáveis por políticas, profissionais da saúde e pacientes.

Every day there is criticism about lack of evidence on traditional, complementary, and integrative medicine (TCIM). This lack of evidence was a common topic in the narratives of over 15 000 health care professionals (1). There is a lot of work to be done on body-mind medicine and many challenges persist (2). But is this narrative evidence-based? Are we really lacking research on TCIM? Or are we just not looking correctly at the evidence?

In 2019, the World Health Organization (WHO) Global Report on Traditional and Complementary Medicine (3) revealed that Member States considered the “lack of research data” in the field the main challenge to advancing regulation processes to integrate TCIM practices into health systems and services. This perception is frequently based on lack of knowledge of existing evidence, on barriers to access (language of publication, paid access), and on difficulties in interpreting results and the research particularities of the field.

Since 2002, the WHO Traditional Medicine Strategy (4) has been encouraging and strengthening the insertion, recognition, and use of TCIM products and practitioners in national health systems at all levels: primary health care, specialized care, and hospital care.

Overall, TCIM evidence production has increased significantly over the last 20 years. During the regional meeting “Advancing towards Universal Health, Contributions of Traditional and Complementary Medicine,” organized by the Pan American Health Organization (PAHO) in 2017 in Managua, Nicaragua, the PAHO/WHO Latin American and Caribbean Center on Health Sciences Information (BIREME/PAHO/WHO) presented a proposal for creation of a Virtual Health Library (VHL) specialized in TCIM. During the meeting, delegates from various countries proposed the creation of a TCIM Collaborative Network for the Americas as a technical cooperation response to the demand from PAHO Member States for reliable sources of scientific evidence on TCIM (5).

Currently there are over 1 million bibliographic references in the VHL TCIM (https://mtci.bvsalud.org/en/). The sheer number of studies creates a challenge for anyone navigating this saturated field. The VHL TCIM is a thematic virtual library that aims to promote the visibility, access, use, and publication of scientific, technical, and educational content that will contribute to promotion, development, and integration of TCIM into health care services and systems in the Americas through stakeholder collaboration.

As a contribution to facilitate access to available evidence and identify knowledge gaps, and in response to a demand from the Ministry of Health of Brazil, BIREME/PAHO/WHO, in collaboration with the Brazilian Academic Consortium for Integrative Health (CABSIN), brought together a group of researchers and experts in the field to develop clinical evidence maps on TCIM. The objective of this article is to present the six steps of evidence map methodology and recently published TCIM evidence maps, including one related to COVID-19.

## MATERIALS AND METHODS

Evidence mapping allows different types of evidence integration, as well as graphical or dynamic representations, through interactive online databases, which facilitates interpretation of results and may also include information about quality analysis and effects (6). These characteristics make it a useful instrument to inform decision-making for managers, health care professionals, and patients and for prioritizing research needs in order to address knowledge gaps.

The first map developed by BIREME/PAHO/WHO was about the clinical effectiveness of ozone therapy, with a medical focus, revealing a need to widen its use. After this experience, the organizations involved in the project deliberated about the areas for the following maps, considering their application in the Brazilian health system. The seven practices selected are included in the National Policy of Integrative and Complementary Practices prioritized by the Ministry of Health of Brazil: traditional Chinese medicine (acupuncture, auriculotherapy, mind and body practices), medicinal plants and phytotherapy, yoga, meditation, reflexology, oral ozone therapy, and shantala (infant massage). Because of the large number of studies in each area, the working groups decided only to include systematic reviews about each of the practices in these evidence maps.

We report the methods and results according to PRISMA guidelines (7) and the International Initiative for Impact Evaluation (3iE) Evidence Gap Methodology (8). We applied AMSTAR 2 (9) to analyze the quality of the included systematic reviews. Each evidence map was supported by a technical expert panel of librarians, practitioners, policymakers, and researchers. The maps are easy to navigate and most of the included studies are free access and with full text available in English.

TCIM evidence map methodology can be described considering six steps, each with a set of activities (Table 1).

## RESULTS

TCIM evidence maps are all available from the TCIM Evidence Map website https://mtci.bvsalud.org/en/evidence-map. These maps include over 800 systematic reviews, 500 interventions, and 400 outcomes (e.g., depression, anxiety, quality of life, stress, sleep quality, physical functions, pain relief, blood pressure, blood glucose, fatigue, balance, humor). Table 2 summarizes the TCIM evidence maps and the number of systematic reviews in each category of outcomes.

In relation to level of confidence, we applied AMSTAR 2 and found studies with high, moderate, and low levels of confidence. Regarding evidence quality, the majority of TCIM evidence maps presented positive effects—although lack of effect was also attributed, as well as a few negative effects—analyzed using different methods by each systematic review. All this information is available on the TCIM Evidence Map website and is easy to access for policymakers, health professionals, and patients.

Due to the challenges and urgency brought by the development of the COVID-19 pandemic, the Ministry of Health of Brazil, BIREME/PAHO/WHO, CABSIN, and the TCIM Network teamed up to develop a specific evidence map to describe different TCIM interventions and report health outcomes that could help in times of COVID-19. The main objective of this evidence map was to systematize scientific evidence in order to support professionals, managers, and researchers in the construction of documents and actions related to TCIM in the response to the pandemic.

This evidence map includes 126 controlled clinical studies and reviews that are arranged in a graphic platform and distributed across 62 interventions and 67 clinical outcomes divided into immunological response, complementary clinical management, and mental health (10).

Until now, there is no evidence of specific treatments for COVID-19. This work synthesized evidence for researchers and health professionals duly trained in the use of complementary therapies in the management of symptoms, especially in the dimensions of mental health and mild symptoms. Most antiviral activity findings described refer to respiratory viruses in general, not specifically to SARS-CoV-2; these may guide new research but do not necessarily support a therapeutic protocol recommendation (10).

## DISCUSSION

Evidence maps represent a broad grouping and repository of evidence published in indexed scientific journals, from various countries, to contribute to evidence-informed practices and also to respond to the ongoing pandemic. There is much evidence about TCIM that still needs to be gathered.

There are several different ways to do evidence mapping (6), but usually the data are presented without showing their effects or “what works,” making it difficult to access all databases and usually not answering specific questions that policymakers, health practitioners, and patients might have.

The aim of making all TCIM evidence maps available through an interactive platform at the VHL TCIM is to facilitate policymakers, health practitioners, and patients’ access to this information. In each map, it is possible to access different interventions, outcomes, and effects in order to promote TCIM evidence-based decision-making. Every map presents the number of studies in each category, areas with gaps, and level of confidence (based on AMSTAR 2) for each included study, so researchers can also identify areas that still require research.

**TABLE 1.** Six-step TCIM evidence map methodology

Step	Activities
***1 Search****. Bibliographic search for TCIM scientific evidence must be carried out in electronic databases and complemented by manual search and/or indication of TCIM specialists collaborating on the project.*	Identification and selection of information sources (databases and journals) that will be consulted; Development of electronic search expressions according to the selected databases and the selected TCIM practice; Conducting manual search and/or indicating revisions in TCIM, not identified in electronic search; Metadata exportation of bibliographic records retrieved in the bibliographic search (electronic and manual); Importing metadata from bibliographic records into a bibliographic reference manager (e.g., Endnote, Mendeley); and Documentation with a detailed description of all bibliographic searches performed.
***2 Selection****. Scientific evidence (scientific articles) identified in the bibliographic search will be evaluated according to previously defined criteria.*	Analysis of the articles identified in the first stage to confirm compliance with the predefined inclusion criteria; Classification of selected evidence in pairs, preferably with blinding, using software (e.g., Rayyan); Evaluation and classification of studies by quality criteria (e.g., AMSTAR 2 in case of systematic reviews).
***3 Categorization****. Data from the studies included in the previous stage will be extracted considering previously defined categories.*	Definition of intervention categories (depending on each TCIM practice) and outcomes (e.g., mental health, pain, cancer) that will be used to map selected review studies; Data extraction such as: Full Text (website link); Citation (complete reference), Population; Database ID; Focus Country (countries in which studies were carried out); Publication Country (country in which the article was published); Publication Year; Analysis about included studies considering information provided by authors and quality criteria: Effect (positive, potential positive, inconclusive, potential negative, negative); Level of confidence (high, medium, low); Type of Review (systematic review, narrative review, scoping review, mixed methods review, protocol, meta-analysis, metasynthesis, etc.); Review Design (clinical studies, randomized controlled studies, observational studies, mixed studies, etc.); Study Design (effectiveness, safety, cost). Review and adjustment of categories.
***4 Informetrics.*** *Development of informetric analysis takes place from metadata of review studies identified and registered in the databases.*	Definition of indicators that will be generated for informetric analysis; Performing the extraction, transformation and loading processes for metadata of the selected studies; Development of visualizations of informetric data to support analysis of the defined indicators; and Integrate visualizations of informetric data in evidence maps and/or graphs from Tableau software.
***5 Evidence map****. Creation and publication of evidence maps consists of graphically representing the evidence found, analyzed, and categorized, in addition to linking with bibliographic records and full texts (when available) of evidence studies.*	Identification of main contents (highlights) to produce maps and/or graphics; Creation of graphic design elements of maps and/or graphics; Inclusion of content in maps and/or graphics with links to bibliographic records in the corresponding databases; and Publication of maps and/or graphics in the VHL TCIM Americas and other selected portals.
***6 Gaps****. Based on analysis of the studies identified and analyzed, a report or scientific article should be organized in order to demonstrate evidence gaps and redundancy (multiple studies on similar issues) related to TCIM, contributing to establishment of national research priorities among research agencies and researchers.*	Definition of analysis criteria for the studies identified and selected for TCIM; Quantitative analysis of studies identified and selected using the defined criteria; Preparation of reports pointing out evidence and redundancy gaps for TCIM; and Publication of reports.

**TABLE 2.** TCIM evidence maps, number of systematic reviews, and principal category of outcomes

	Physical and Metabolic Effects	Mental Health	Vitality, Well-Being and Quality of Life	Socio-Environmental and Spiritual
Acupuncture	139	19	10	10
Auriculotherapy	28	9	7	4
Medicinal Plats and Phytotheraphy	80	21	24	0
Meditation	53	137	72	10
Mind and Body Pratices from TCM	93	36	105	7
Oral Ozone Therapy	15	3	13	5
Reflexology	17	10	11	0
Shantala	20	27	33	17
Yoga	84	67	79	28

In these times of uncertainty, we would like to have all the cause-effect answers that Western medicine promises (11), as well as access to all the hard technology needed to attend severe COVID-19 cases and to protect health care workers. But alongside these we can use the abundant TCIM evidence to provide information to help people isolate at home. For those at risk of developing or deteriorating mental health issues, chronic disease, or pain, the evidence shows benefits and possible uses for TCIM.

### Limitations

We understand that evidence maps can only provide a broad overview of research, and the TCIM evidence maps cited will not be able to answer more refined questions, such as the most adequate TCIM application method, differences between health services, adequate training for practitioners, patient access, and self-application effects. But we believe future research, including qualitative research and case studies, is needed to answer these questions and is very important for TCIM-related evidence development.

### Conclusion

Evidence maps provide an easy visualization of valuable information on TCIM for policymakers, health practitioners, and patients, in order to promote evidence-based complementary therapies in line with the WHO Traditional Medicine Strategy 2014–2024 (3).

## Disclaimer.

Authors hold sole responsibility for the views expressed in the manuscript, which may not necessarily reflect the opinion or policy of the *RPSP/PAJPH* and/or PAHO.
